# Identification and functional validation of a new gene conferring resistance to *Soybean Mosaic Virus* strains SC4 and SC20 in soybean

**DOI:** 10.3389/fpls.2024.1518829

**Published:** 2025-01-27

**Authors:** Muhammad Muzzafar Raza, Huiying Jia, Muhammad Khuram Razzaq, Bowen Li, Kai Li, Junyi Gai

**Affiliations:** Soybean Research Institute & MARA National Center for Soybean Improvement & Ministry of Agriculture and Rural Affairs (MARA) Key Laboratory of Biology and Genetic Improvement of Soybean (General) & State Innovation Platform for Integrated Production and Education in Soybean Bio-breeding & State Key Laboratory for Crop Genetics and Germplasm Enhancement & Jiangsu Collaborative Innovation Center for Modern Crop Production, Nanjing Agricultural University, Nanjing, Jiangsu, China

**Keywords:** Mendelian inheritance, Soybean Mosaic Virus, single nucleotide polymorphism, resistance, linkage mapping

## Abstract

Soybean Mosaic Virus (SMV) poses a serious threat to soybean production, often resulting in considerable yield losses or complete crop failure, particularly if infection occurs during early growth stages. While several SMV resistance genes have been identified, the genetic basis of resistance to certain strains remains poorly understood. Among the 22 SMV strains, SC4 and SC20 are considered pathogenic in Central China. Dominant genes resistant to SC4 (*Rsc4*) on Chr.14 in Dabaima and to SC20 (*Rsc20*) on Chr.13 in Qihuang-1 have been identified. Kefeng-1 is resistant to SC4 and SC20. This study aimed to determine whether the resistance to SC4 and SC20 in Kefeng-1 was identical and whether *Rsc4* and *Rsc20* in Dabaima and Qihuang-1 are also present in Kefeng-1 due to translocation. Mendelian experiments using F_1_, F_2,_ and recombinant inbred lines (RIL_3:8_) of Kefeng-1 (resistant) and NN1138-2 (susceptible) indicated a single dominant gene inheritance pattern in SC4 and SC20, respectively. Linkage mapping showed two loci for SC4 and SC20 in neighboring single nucleotide polymorphism linkage disequilibrium blocks (SNPLDB) marker regions of 253 kb and 375 kb, respectively, in Kefeng-1. Association between SNPs in possible gene regions of Kefeng-1 and resistance data showed SNP11692903 jointly as the most significant SNP, exhibiting the highest *χ^2^* value. By comparing SNP11692903 to possible gene sequences in the coding region, *Glyma02g13380* was identified as a joint candidate gene. The results were validated using qRT-PCR, virus induced gene silencing (VIGS), and gene-sequence. Therefore, the two Mendelian genes on chromosome 2 in Kefeng-1 responsible for SC4 and SC20 resistance are unique genes, different from *Rsc4* in Dabaima and *Rsc20* in Qihuang-1. Hence, one gene is involved in resistance toward two SMV strains resistance. This result challenged our previous hypothesis of a single dominant gene responsible for resistance against a single strain and underscored the potential for using multiple resistance sources aimed at enhancing SMV resistance in breeding practices.

## 1 Introduction

Soybean is an economically vital grain legume because of its protein and edible oil of excellent quality that are used in industry and routine human affairs. One of the major risks for soybean production in China and worldwide is *Soybean Mosaic Virus* (SMV). Viral infection during the early growth stages of crops may lead to maximum yield loss or complete crop failure (Hill, 2003). If infection occurs within three weeks of sowing, it can cause up to 100% crop failure (Hill and Whitham, 2014).

Several SMV strains have been reported worldwide; however, the pathogenic relationship among these strains has not yet been completely elucidated. In Japan, five strains, represented by A, B, C, D, and E, have been reported (Takahashi et al., 1980). In the USA, seven strains (G1, G2, G3, G4, G5, G6, and G7) were identified by Cho and Goodman (1982), and this grouping was modified by Chen and Choi (2007). Three discrete resistance loci for these strains on Chromosome 2, 13 and 14 from their natural sources have been discovered (Jeong and Saghai Maroof, 2004). In China, 22 SMV strains (SC1-SC22) have been identified in ten varieties of/differential hosts (Li et al., 2010; Wang et al., 2013; Zhi et al., 2005). Host plant resistance (HPR) is a cost-effective and eco-friendly technique for combating SMV. Numerous sources of SMV resistance have been identified in soybeans, most of which are resistant to a few strains. In most cases, the resistance is conferred by a single dominant gene. Until now, many self-governing resistance loci with diverse SMV strain specifications have been recognized. To date, three autonomous loci; *Rsv1*, *Rsv*3, and *Rsv*4 in the USA and several *R_SC_* loci in China have been reported and identified that confer SMV resistance (Liu et al., 2016). However, only a few resistance genes have been cloned, and their characteristics are yet to be identified.

A lot of research on SMV resistance has been conducted worldwide such as in cultivar “Heinong 84” a single dominant gene on Chr.13 which is allele of *Rsv1* is controlling resistance against strain N3 (Li et al., 2022). Similarly, another SMV resistant novel locus *R_smv_-11* for SC7 was identified (Zhang et al., 2022). *Rsv1*, *Rsv3* loci were discovered in Egyptian soybean cultivars, SMV SC3 was studied in NN1138-2 and Parasite Driven Resistance (PDR) for SMV was studied (Eid et al., 2023; Liu et al., 2023; Yang et al., 2024).

One of the basic requirements for the development of SMV-resistant soybean cultivars using molecular breeding techniques is the identification of genomic regions with the most probable candidate disease-resistant genes. Linkage mapping is an excellent technique for gene identification, especially when combined with map-based cloning (Fu et al., 2006; Hayes et al., 2004; Hwang et al., 2006; Li et al., 2006), but the accuracy of this technique depends on map resolution. Recently, single-nucleotide polymorphism (SNP) markers have become popular because they focus on molecular breeding and are suitable for high-throughput recognition setups and platforms (Ganal et al., 2009). SNP markers are commonly used to develop high-quality resolution maps. These SNP markers are common sources of variability in the genome and are used to measure genetic diversity and population structure. Different public databases comprise extensive information on SNPs in numerous plant species, including rice, wheat, maize, and soybean (Akond et al., 2013; Cavanagh et al., 2013; Ganal et al., 2011; Jones et al., 2009; McNally et al., 2009; Rostoks et al., 2005; Song et al., 2013; Tian et al., 2015).

Among the 22 SMV strains, SC4 and SC20 are moderately pathogenic strains prevalent in the Huang-Huai and Yangtze River Valleys in China (Li et al., 2010). *R_SC_*_4_ locus has been previously reported in Dabaima and contains three candidate resistance genes on chromosome (Chr.) 14 (Wang et al., 2011). In contrast, *R_SC_*_20_ has been reported on Chr.13 in Qihuang-1 (Karthikeyan et al., 2018) as a single dominant resistance gene that has not yet been cloned. Based on these and previous studies, we recognized that a single dominant gene is responsible for single-strain resistance. Recently, we found that Kefeng-1 also shows resistance against SC4 and SC20; however, the responsible elements are not located on Chr.14 and Chr.13 (Wang et al., 2011). Therefore, this study aimed to investigate whether the resistance to SC4 and SC20 in Kefeng-1 are conferred by distinct genes, determine their location, and clarify whether the newly found genes resistant to SC4 and SC20 were distinct or identical to *R_SC_*_4_ and *R_SC_*_20_ in Dabaima and Qihuang-1 due to translocation. The objectives of this study were to identify resistance genes for both strains in Kefeng-1 using the techniques of Mendelian inheritance, gene mapping, gene fine mapping, quantitative reverse transcription polymerase chain reaction (qRT-PCR), virus-induced gene silencing (VIGS), and gene sequencing to gain a thorough understanding and improve our resistance breeding strategy (Zhan et al., 2006).

## 2 Materials and methods

### 2.1 Plant genetic materials

The soybean cultivar NN1138-2 was susceptible (S), and Kefeng-1 was resistant to the SMV strains SC4 and SC20. To investigate the inheritance patterns of resistance, F_1_, F_2,_ and RIL_3:8_ (comprising 427 RILs) derived from a cross between Kefeng-1 and NN1138-2 were used. For fine mapping of candidate resistance genes, only the RIL population was used. The parental lines were crossed to acquire F_1_ at the Dangtu Research Station, National Center for Soybean Improvement (NCSI), Nanjing Agricultural University, China. The F_1_ plants were self-fertilized to obtain F_2_ plants in Hainan province. To derive the RIL population, a single-seed descent procedure was used to obtain the F_3:8_ generation. Both parents and their derived populations were cultivated for screening in plastic pots (w20×h20 cm) in a fully controlled, conditioned glass house at the Baima Agriculture Station, Nanjing Agricultural University, China.

### 2.2 Preparation and maintenance of SMV inoculum

The SMV strains SC4 and SC20 were supplied by NCSI. The SMV isolates were maintained separately on NN1138-2. Plants at the first true-leaf stage were successively inoculated under controlled conditions in an aphid-free glass house. Inoculation performed mechanically as described earlier (Che et al., 2020). Infected fresh leaves of NN1138-2 were ground using a pestle in a mortar containing 0.01 mol/L of sodium phosphate buffer (4–5 ml/g of leaf tissue; 7.2 pH) to prepare the inoculum. The mortar containing the inoculum was placed on ice during inoculation. Before inoculation, the leaves were dusted with 600 mesh carborundum powder as an abrasive and rinsed with tap water after inoculation. The SC4 and SC20 inocula were verified by the real inoculation of a set of ten differential soybean hosts, as described by Li et al. (2010) (**Supplementary Table 1**).

### 2.3 Inoculum application and disease assay

The plants of F_1,_ F_2,_ and RIL populations with their parental lines (ten plants per line) were mechanically inoculated separately with SC4 and SC20 inoculums at the true-leaf unfolding stage (seven-to-ten days after sowing). The pathological response was recorded based on optical observance with a time interval of 10 days until 40 days after inoculation as described by Karthikeyan et al. (2017). Mosaic and necrosis-like symptoms were observed on newly emerging leaves after inoculation. Categorization criteria for susceptibility and resistance to specific SMV strains were established based on symptomatic and symptomless plants. Plants that did not develop any salient evidence of infection after inoculation and looked like uninoculated plants and those with flimsy necrotic stripes, minor necrotic lesions, and fainted mosaic evidence with less than 10% rate of incidence were characterized in the resistance category, whereas plants that displayed symptoms such as mosaic with or without necrotic leaves and general necrosis with an incidence rate greater than 10% were regarded as susceptible.

### 2.4 Statistical analysis

The chi-square test was used to test the phenotypic assessment of segregation in the F_2_ and RIL populations for goodness of fit to the Mendelian ratio. The values of Chi-square and *p* were obtained using the SAS software (Version 9.4).

### 2.5 Genotypic resequencing and gene mapping

Genotypic data of the RIL population (Kefeng-1×NN1138-2) comprising 3683 SNPLDB markers were used to track the resistant genomic regions provided by NCSI. The construction procedure for the SNPLDB markers has been outlined by He et al. (2017). The protocol is described in brief as follow: Genomic DNA from both parents and the RIL population was isolated from young leaves following the cetyltrimethylammonium bromide (CTAB) methodology (Murray and Thompson, 1980). All sequence libraries were constructed using the Illumina HiSeq 2000 protocol. The SNP calling and genotyping were performed using component combining Burrows-Wheeler Aligner (Li and Durbin, 2009) and Sequence Alignment/Map tools (SAMtools) (Li et al., 2009). SNPs with deletions of less than or equal to 20%, error rates of less than or equal to 1%, and heterozygosity rates of less than 5% were deleted, and NPUTE was used to fill in the SNPs (Roberts et al., 2007). An SNPLDB marker was structured with multiple haplotypes as alleles by grouping the SNPs within an LD block. Each genotype was resolved using related haplotypes at each locus. The LD values between multi-allelic SNPLDBs were calculated as previously described by Farnir et al. (2000).

Linkage map construction was performed using JoinMap 4.1. A total of 3683 SNPLDB markers distributed among all 20 chromosomes of soybean were used to construct a linkage map. The segregation of markers on each chromosome was 243 on Chr.1, 157 on Chr.2, 198 on Chr.3, 280 on Chr.4, 126 on Chr.5, 112 on Chr.6, 114, 225, 170, 267, 134, 75, 157, 216, 167, 206, 197, 322, 167 and 13 on Chr. 7, 8. 9, 10, 11, 12, 13, 14, 15, 16, 17, 18, 19 and 20 respectively. A logarithm of Odds (LOD) threshold of 3.0 was used, and the marker distance was determined according to the Kosambi function (Kosambi, 2016).

### 2.6 Selection of candidate gene and association analysis

After genomic region identification, the total number of genes located in that region were selected from the Williams 82 reference genome (GlymaWm82.a1.v1), and homologous genes in *Arabidopsis thaliana* with their functional annotation were identified from soybase (https://www.soybase.org) and phytozome (https://www.phytozome.jgi.doe.gov) database. The whole-genome resequencing (WGRS) data from 427 families were used to accumulate the total SNPs in the identified genomic region. Genes possessing SNPs in their coding regions with nonsynonymous translations were selected. Finally, WGRS gene marker data were used to find the most plausible candidate gene, and the affiliation between gene markers and the phenotypic data set of all 427 RILs was inspected by the Chi-square test using software *R 3.2.3* at *p* < 0.01. We used the most probable SNP in the coding region to identify the most probable gene in the shortened region.

### 2.7 SC4 and SC20 resistance locus identification using WGRS gene-marker data

To obtain the most appropriate SC4 resistance gene, WGRS gene marker data from 427 families were used to construct a linkage map to identify SC4 resistance loci among the gene marker loci. Similarly, another linkage map was constructed for SC20.

### 2.8 qRT-PCR for expression analysis

Quantitative RT-PCR was performed to determine the candidate gene expression. TaqMan software (http://www.genscript.com) was used to design gene-specific primers from the respective coding sequences acquired from the SoyBase database (http://www.soybase.org/) (**Supplementary Table 2**). Tubulin was used as the internal reference gene. The expression differential of genes was quantified by the 2^–ΔΔ Ct^ method (Livak and Schmittgen, 2001). Each biological replicate contained three samples with three technical replicates.

### 2.9 VIGS vectors construction

After identifying candidate resistance genes, their functions were validated using VIGS. Bean Pod Mottle Virus (BPMV) BPMV-silencing vectors were constructed and inoculated as previously described (Zhang et al., 2009). The restriction enzymes *BamH*I and *Sal*I (NEB, Beijing, China) were mixed with pBPMV-IA-V2-R2, and a 300 bp region of all candidate genes (*Glyma02g13230, Glyma02g13380, Glyma02g13401, Glyma02g13470, Glyma02g13570*, and *Glyma02g13630*) was amplified from Kefeng-1. The BPMV-linearized vectors and gene fragments were combined using recombination homology. The recombinant plasmids and pBPMV-IA-R1M were separately multiplied and mixed in equal ratios to mechanically inoculate the NN1138-2 plants.

### 2.10 Silencing efficiency estimation and SC4 and SC20 accumulation detection by qRT-PCR and ELISA

NN1138-2 leaves showing SMV symptoms after inoculation with silencing vectors were used for RNA extraction. Total RNA extraction and cDNA synthesis were performed according to the manufacturer’s protocols (Accurate Biotechnology Co., Ltd., Changsha, China; Vazyme Biotech, Nanjing, China). RT-PCR was performed using specific primers to confirm the insertion of the gene fragments. After confirmation of the inserted gene fragment, diseased leaves with silencing vectors for the candidate genes were inoculated on the first true leaves of Kefeng-1, and SMV strains SC4 and SC20 were inoculated separately on the first trifoliate leaves. Ten days after SMV inoculation, upper uninoculated leaves were collected for the detection of the silencing efficiency of the concerned vectors and SMV accumulation by qRT-PCR and double-antibody sandwich enzyme-linked immunosorbent assay (DAS-ELISA). The expression differential of respective genes was quantified using the 2^−ΔΔCt^ method. Each biological replicate comprised three samples with three technical replicates. The primers used for gene fragment insertion confirmation, silencing efficiency, and CP content are listed in **Supplementary Table 3.**

## 3 Results

### 3.1 Mendelian inheritance mechanisms of resistance toward SC4 and SC20 in Kefeng-1

Both parents, Kefeng-1 and NN1138-2, and their offspring were inoculated with SMV strains SC4 and SC20, which produced different symptoms. Kefeng-1 did not show any symptoms in response to either SMV strain, and the plants were similar to the non-inoculated plants in the control group. In contrast, plants of the second parent NN1138-2 showed stunted growth symptoms, reduced leaf size, and mosaic patterns on their leaves within 15–20 days post-inoculation (dpi) with SMV strains. Consequently, Kefeng-1 and F_1_ generations were resistant to SC4 and SC20, whereas NN1138-2 was susceptible.

Polymorphisms were observed in both parents (Kefeng-1 and NN1138-2) after inoculation with SMV strains SC4 and SC20. All plants of the sensitive parent NN1138-2 were scrawny and scrubby in growth, with mosaic symptoms on their leaves, whereas immune parent plants remained symptomless, similar to uninoculated control plants, until 30 days of inoculation. F_1_s of Kefeng-1 and NN1138-2 showed excellent immunity to SC4 and SC20, as evident from the dominant resistance pattern recorded in Kefeng-1. In total, 179 F_2_ plants and 427 RILs obtained from parental crosses were inoculated with SC4. The phenotypic segregation ratio was 133R:46S (fitting 3:1 ratio) in F_2_ whereas the phenotypic and genotypic ratios were 211R:216S (fitting1:1 ratio) in RILs, indicating the involvement of a single dominant gene in SC4 resistance (**Table 1**). Similarly, 179 F_2_ plants and 411 RILs were inoculated with SC20, which showed a phenotypic ratio of 133R:46S (fitting 3:1 ratio) in F_2_ plants and 204R:207S (1:1 ratio) in RILs, with another dominant gene responsible for resistance to SC20 in Kefeng-1 (**Table 1**).

**Table 1:** **Mendelian inheritance of SC4 and SC20 resistance in parents, F_1_, F_2_, and RILs** .

	SC4						SC20					
Parent/progeny	No. of plants/lines		Total	Expected ratios	$\chi^{2}$	*p*-Value	No. of plants/lines		Total	Expected ratios	$\chi^{2}$	*p*-Value
	R	S					R	S				
Kefeng-1	20	–	20				20	–	20			
NN1138-2	–	20	20				–	20	20			
F_1_	25	–	25				10	–	10			
F_2_	133	46	179	3:1	0.0466	0.8292	133	46	179	3:1	0.0466	0.8292
RILs	211	216	427	1:1	0.0585	0.8088	204	207	411	1:1	0.0219	0.8824

R for resistant, and S for susceptible.

### 3.2 Mapping the genes conferring resistance to SC4 and SC20 using linkage analysis with SNPLDB as markers

**Figure 1:**
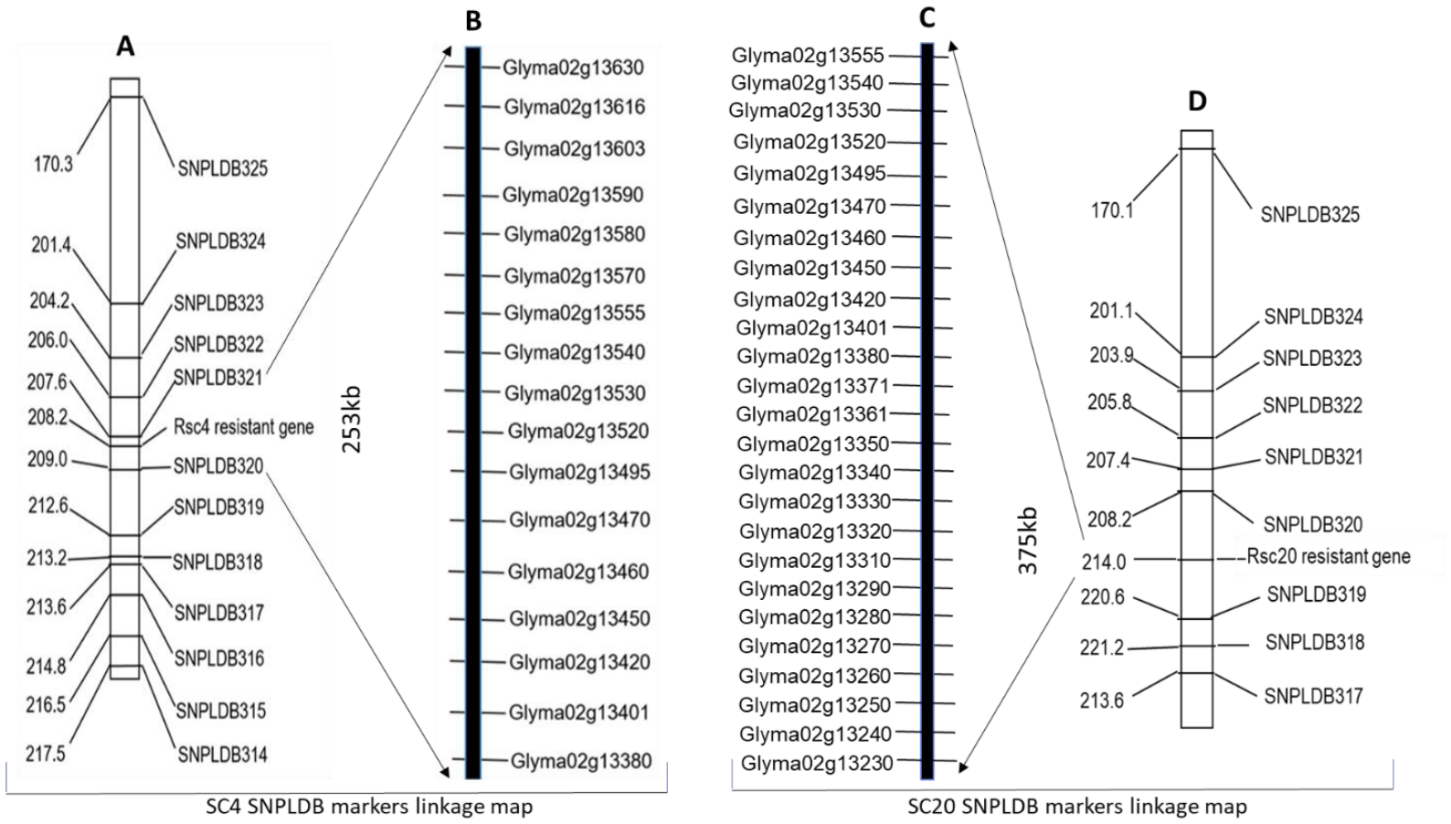
**(A, D)** SNPLDB linkage map showing SC4 and SC20 resistant loci between SNPLDB320-SNPLDB321 and SNPLDB319-SNPLDB320 on chromosome 2 constructed by WGRS genotypic and phenotypic data of 427 recombinant inbred lines, indicating genetic distance left side in centi Morgan (cM) and right-side names of SNPLDB markers, **(B, C)** genes found in 253-kb and 375-kb identified genomic regions retrieved from soybean William-82 reference genome GlymaWm82.a1. v1 in SoyBase (http://www.soybase.org).

We investigated the SC4 and SC20 resistance-related genomic regions, with an RIL population of 427 families. Two separate linkage maps covering all twenty chromosomes of soybean were constructed based on the phenotypic and genotypic data of 427 individual lines with 3683 SNPLDB markers using JoinMap 4.1 at a threshold LOD 3.0. SC4 resistance region was located at 208.2 cM between two SNPLDB markers from SNPLDB320-SNPLDB321 with a genetic position of 209.0–207.6 cM and SC20 was at 214.0 cM between SNPLDB319-SNPLDB320 positioned at 220.6–208.2cM on Chr.2. These SNPLDB markers showed a strong association with adjacent loci conferring resistance to SC4 and SC20, located at the physical positions 11693196–1944513 bp and 11486875–1693196bp with approximate genomic intervals of 253 kb and 375 kb, respectively (**Figure 1**). In accordance with the Williams 82 soybean reference genome GlymaWm82 model 1.1, 17 genes in the SC4 region and 25 genes in the SC20 region with some common genes were situated. Their ornamentation on Chr.2 is shown in **Figure 1**, and the functional annotation of *Arabidopsis* homologous genes is presented in **Supplementary Table 4.**

### 3.3 Candidate gene identification for resistance to SC4 and SC20 in the mapped regions through SNP association

We screened the most likely candidate genes for SC4 and SC20 resistance from all the genes in both regions over four gradual steps. In the first step, the WGRS was performed with the SNP dataset of all RIL families. A total of 26 SNPs in total were remunerated in the preoccupied SC4 and SC20 regions. Only 8 genes comprised 12 out of 26 SNPs, whereas 23 leftover genes did not comprise any SNP. *Glyma02g13230, Glyma02g13361, Glyma02g13371, Glyma02g13401*, and *Glyma02g13470* were each associated with one SNP; *Glyma02g13380* and *Glyma02g13570* were linked with two SNPs, whereas *Glyma02g13630* possessed three SNPs (**Table 2**).

**Table 2:** **Eight SC4 and SC20 resistance nominee genes with their genomic data referred to William82 reference genome (GlymaWm82.a1. v1,** http://www.soybase.org).

Sr. No	Gene	Gene Position	No. of SNPs	Allele	SNP Position
1	*Glyma02g13230*	11484118-11491037	1	G/A	11486875
2	*Glyma02g13361*	11647493-11663260	1	C/T	11662819
3	*Glyma02g13371*	11670695-11677275	1	T/A	11670897
4	*Glyma02g13380*	11692903-11694668	2	T/A	11693196
				A/G	11693900
5	*Glyma02g13401*	11729655-11746942	1	C/G	11739943
6	*Glyma02g13470*	11779259-11782486	1	T/A	11779670
7	*Glyma02g13570*	11890308-11895815	2	T/G	11893075
				T/G	11893936
8	*Glyma02g13630*	11943213-11849714	3	G/A	11944513
				C/T	11944870
				C/T	11946524

In step two, we examined the coding and non-coding regions of eight SNP-holder genes. For SNP posture on genes, the genomic sequences of the concerned genes were retrieved from the SoyKB database (Soybean Knowledge Base, http://soykb.org). We found that SNPs in *Glyma02g13361, Glyma02g13371*, and *Glyma02g13401* were located in an intron and those in *Glyma02g13470* in a 5′UTR region. One of the two SNPs in *Glyma02g13570* was found in introns and the other in exons. Out of the three SNPs in *Glyma02g13630*, two were located in introns and one in exons, whereas *Glyma02g13230* and *Glyma02g13380* associated with one and two SNPs, respectively, had exons with nonsynonymous features of translation (**Table 3**).

**Table 3:** **Four candidate genes indicating SNP location in their coding region and one gene in 5′UTR verified from SoyKB** ( **http://soykb.org** **).**.

Gene	No. of SNPs	SNP localized Zone	SNP associated CDS region	SNP position
*Glyma02g13230*	1	Exon	11485651-11488802	11486875
*Glyma02g13380*	2	Exon	11693175-11694242	11693196
		Exon		11693900
*Glyma02g13470*		5′UTR	5′UTR	11779670
*Glyma02g13570*	2	Exon	11893401-11894308	11893936
		Intron		11893075
*Glyma02g13630*	3	Exon	11944415-11944581	11944513
		Intron		11944870
		Intron		11946524

**Figure 2:**
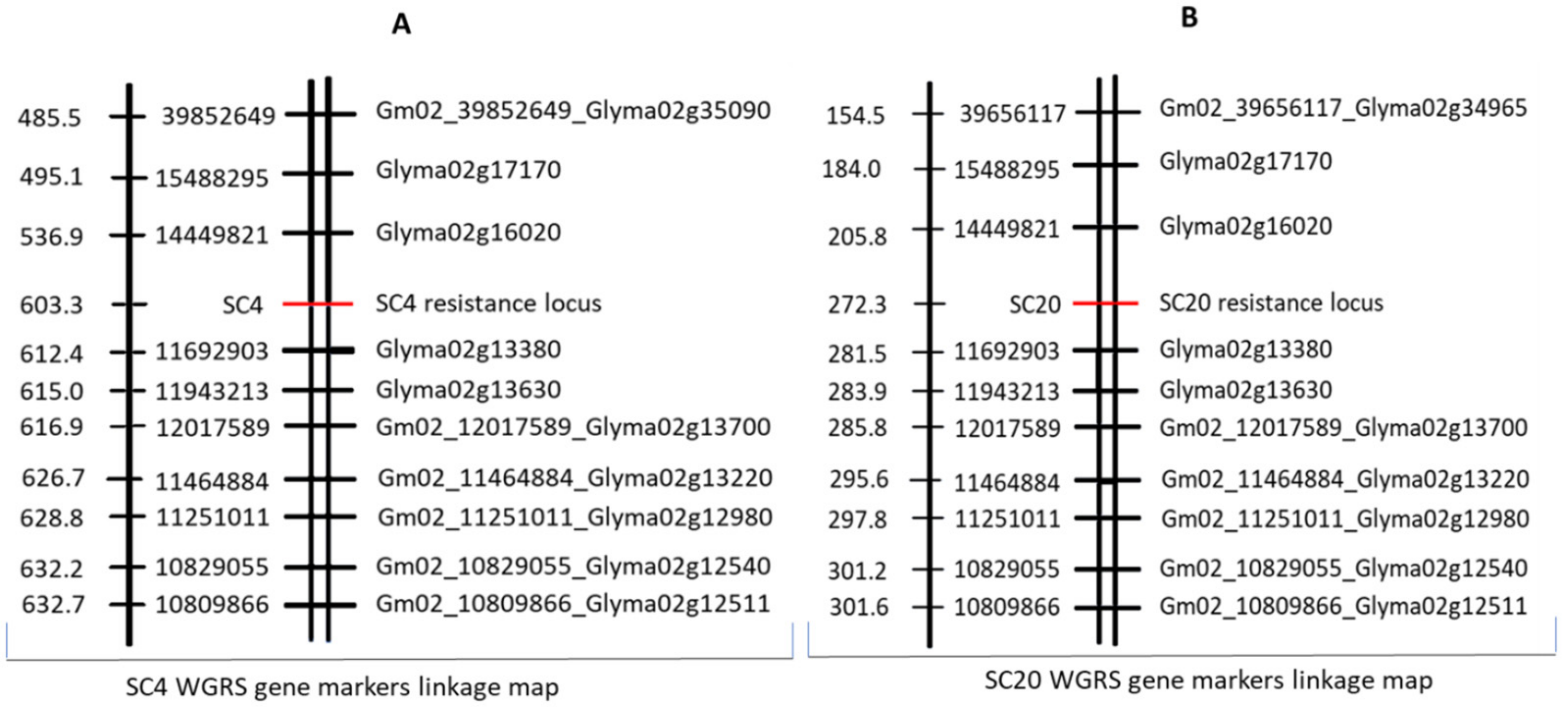
**(A)** linkage map for SC4 and **(B)** for SC20 showing resistant loci between two gene markers loci on chromosome 2 constructed by WGRS gene markers data and phenotypic data for SC4 and SC20 resistance of 427 recombinant inbred lines (RILs) indicating genetic distance in centi Morgan (cM) on its left side, SNP position in its middle, and gene markers on right-side.

Association analysis between gene markers from the WGRS SNPs data and phenotypic data of RILs for SC4 and SC20 resistance was performed in the third step. The results showed that *Glyma02g13630* was significantly correlated with SC4 resistance, *Glyma02g13230* with SC20 resistance, and *Glyma02g13380* and *Glyma02g13470* were significantly associated with both strains (**Table 4**). Finally, in the fourth step, two separate linkage maps covering all twenty chromosomes of soybean were constructed using phenotypic data of 427 individual lines for SC4 and SC20 resistance and WGRS SNP gene markers data with the help of JoinMap 4.1 at a threshold level of LOD 3. Both SC4 and SC20 resistance loci were located between SNP11692903 and SNP14449821 on Chr.2 at 603.3 cM and 272.3 cM, respectively. SNP11692903, within the gene marker *Glyma02g13380*, was the closest to the SC4 and SC20 resistance loci (**Figure 2**). Eventually, based on polymorphisms (2 SNPs) in the coding region, a highly significant correlation with the resistance trait, and location nearest to the resistance loci, *Glyma02g13380* was considered to be the best candidate gene for SC4 and SC20 resistance in Kefeng-1. Furthermore, we hypothesized that the two different single dominant locus controlling SC4 and SC20 resistance, depicted in Mendelian inheritance, was actually the same gene that controlled the resistance of both strains.

**Table 4:** Candidate genes associated with SC4 and SC20 resistance.

Sr. No.	Gene	*D.F*	$\chi^{2}$	*P*-Value	$\chi^{2}$	*P*-Value
			SC4		SC20	
1	*Glyma02g13230*	1	–	–	202.18	2.2e-16
2	*Glyma02g13380*	1	325.71	2.2e-16	297.8	2.2e-16
3	*Glyma02g13470*	1	311.93	2.2e-16	285.42	2.2e-16
4	*Glyma02g13630*	1	305.42	2.2e-16	–	–

### 3.4 Validation of *Glyma02g13380* through expression profile analysis

**Figure 3:**
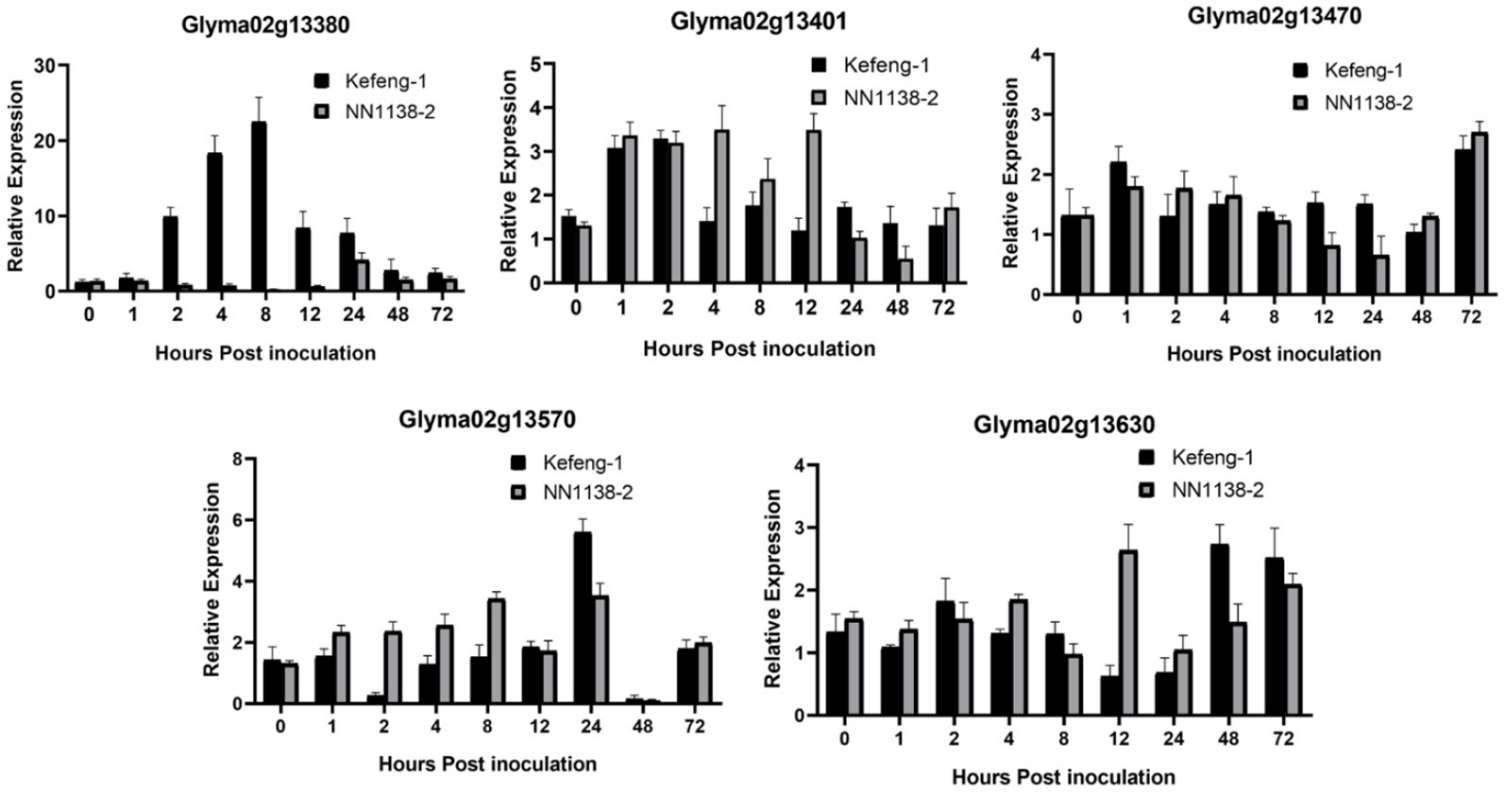
Differential expression profile of candidate genes resistant to SC4. Y-axes represent a relative expression of the genes between samples inoculated SMV and 0.01mol/L of phosphate buffer saline (PBS), and X-axes represent the time scale of sample collection.

**Figure 4:**
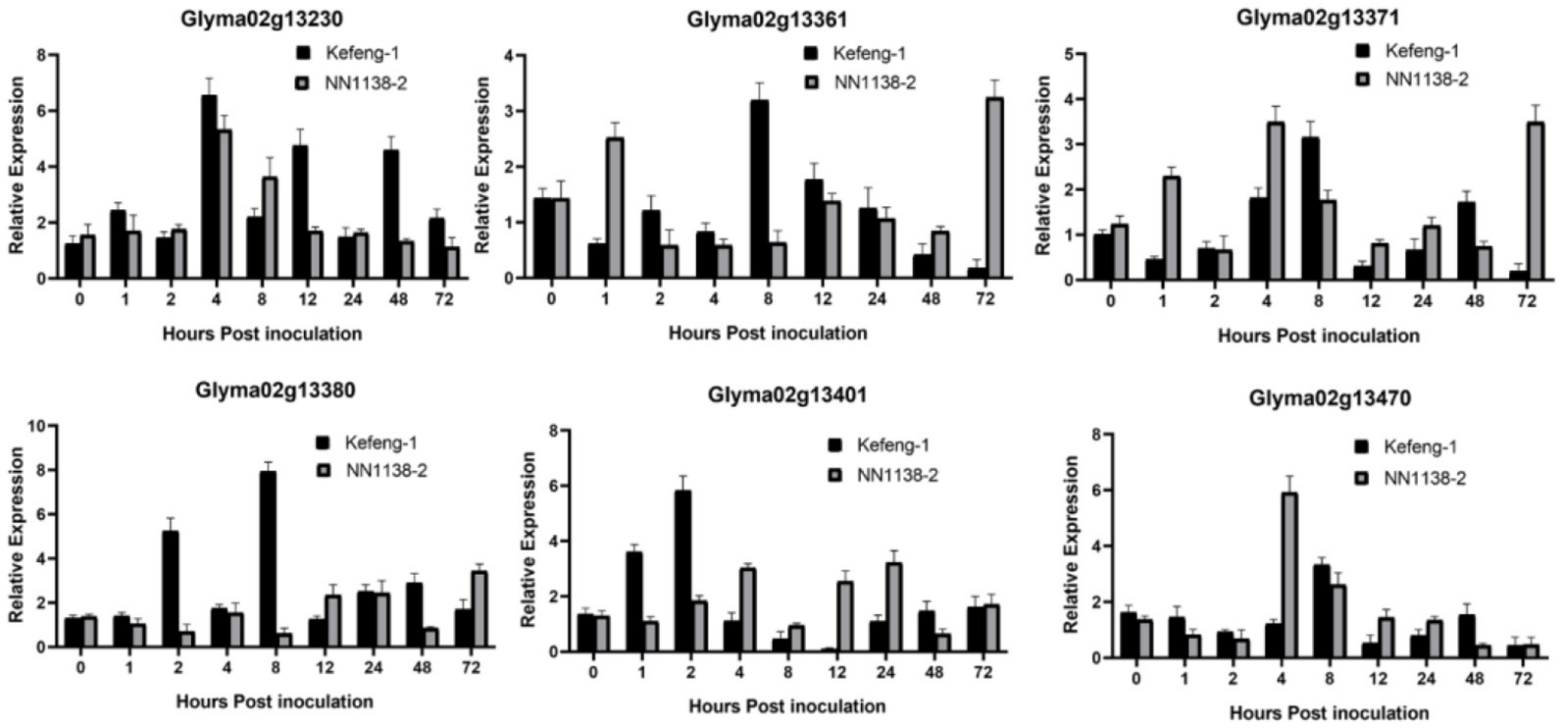
Differential expression profile of candidate genes resistant to SC20. Y-axes represent the relative expression of the genes between samples inoculated with SMV and 0.01mol/L of phosphate buffer saline (PBS), and X-axes represent the time scale of sample collection.

All genes with polymorphisms (five from the SC4 resistance region in **Figure 3**, and six from SC20 with three common genes in **Figure 4**) were assessed for their differential expression. The expression profiles of the genes on the groundwork of SNP enrichment were formulated with the help of quantitative real-time PCR using SC4 and SC20 separately inoculated leaves of both parental genotypes (Kefeng-1 and NN1138-2).

All five genes from the SC4 resistance region behaved differently in the expression analysis. *Glyma02g13380* seemed to exhibit a higher level of upregulated expression between the range of 2.84- to 24.78-folds in the resistant genotype at most time frames, especially at 2, 4, 8, 12, and 24 hours-post inoculation (hpi), whereas it was downregulated in susceptible parents at the same time points, except for 24 hpi (4.81 folds), when it was upregulated. Highly increased expression in the resistant parent was observed at 8 hpi with 24.78 folds, which is the statistically significant highest expression, followed by 19.96 folds at 4 hpi, 10.77 folds at 2 hpi, 9.94 folds at 12 hpi, and 9.07 folds at 24 hpi (**Figure 3**). *Glyma02g13401* and *Glyma02g13470* were expressed equally in both parents at most time points, excluding 4, 8, and 12 hpi in *Glyma02g13401* and 72 hpi in *Glyma02g13470*, where both genes were upregulated in the susceptible parental genotype. In *Glyma02g13570* up-regulation was recorded at 2, 4, and 8 h post-inoculation in the susceptible genotype Nannong 1138-2. In contrast, at 24 hpi, a higher upregulation was observed in the resistant parent Kefeng-1 compared to the opposite parent. This gene was downregulated at 2 hpi in Kefeng-1 and 48 hpi in both parental lines. Similarly, *Glyma02g13630* is not only expressed in the resistant parent but also in the susceptible parent at most time points; a higher upregulation was observed at 12 hpi in the susceptible parent and at 48 and 72 hpi in the resistant parent.

Similarly, the expression of six genes from the SC20 resistance region was observed (**Figure 4**). *Glyma02g13230* was upregulated in both parents at almost every time point, especially at 4, 12, and 48 hpi in the resistant parent and 4 and 12 hpi in the susceptible parent. *Glyma02g13361* and *Glyma02g13371* were expressed at low levels in both parents at 1, 4, 8, and 72 hpi. *Glyma02g13380* was upregulated in the resistant parent with a maximum magnitude of 8.24 folds at 8 hpi followed by 5.65 folds at 2 hpi and 3.18 folds at 48 hpi, whereas it was downregulated in the susceptible parent at 1, 2, 4, 8, and 48 hpi, with little upregulation at 72 hpi (**Figure 4**). *Glyma02g13401* was upregulated at 2 hpi (6.20 folds), followed by 1 hpi (3.79 folds) in the resistant parental line and at 4, 12, and 24 hpi in the susceptible parent. *Glyma02g13470* was upregulated only at 4 hpi (6.3) in the susceptible parents. Considering this, the expression results of SC4 and SC20 resistance genes, *Glyma02g13380* appears to be the most probable candidate gene for both SMV strains, as it is the only gene that shows significant and maximum upregulation in Kefeng-1 (resistant parent) and downregulation in NN1138-2 (susceptible parent) (**Figures 3**, **4**).

### 3.5 Validation of *Glyma02g13380* through VIGS analysis

**Figure 5:**
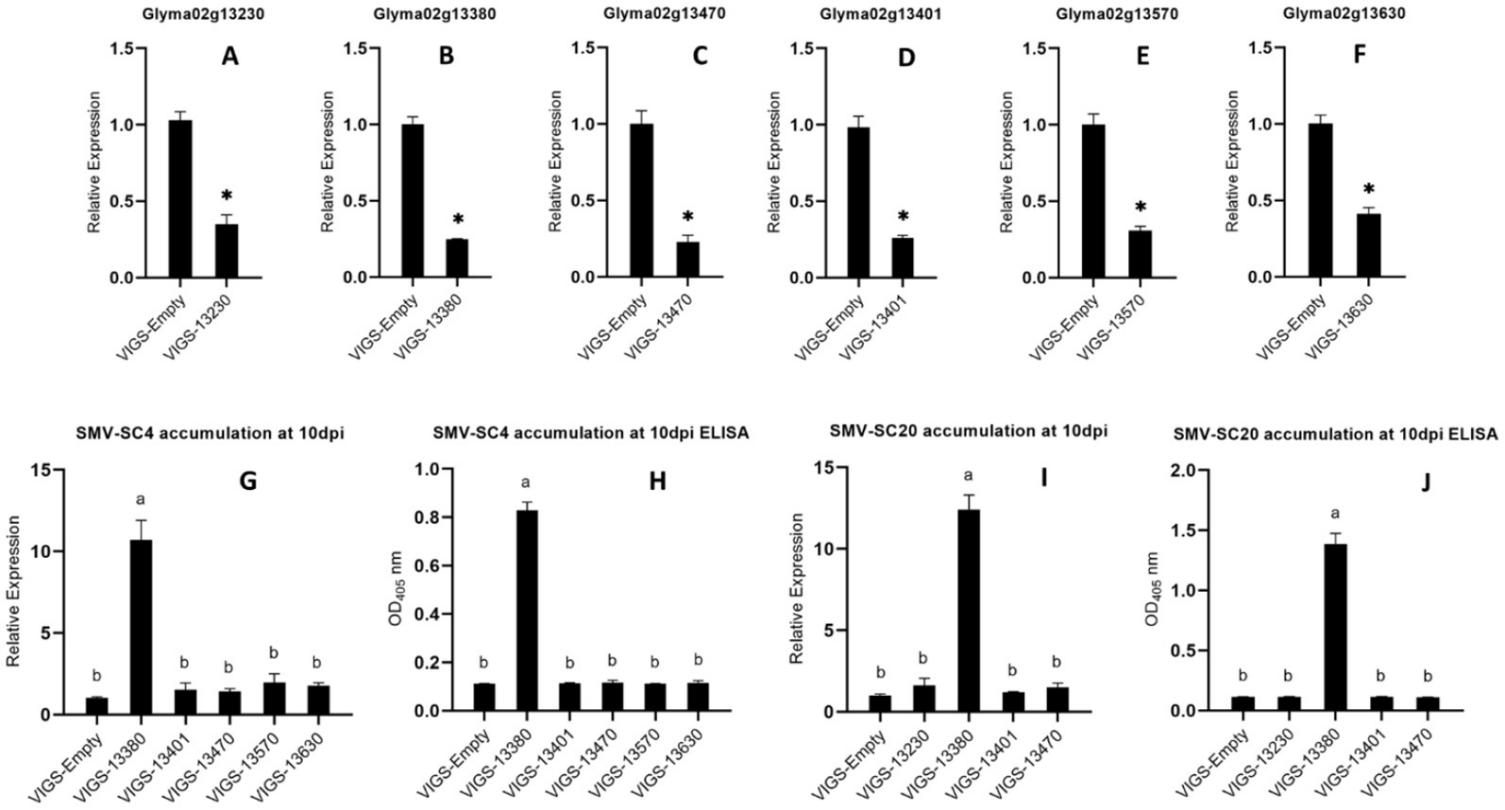
**(A–F)** Silencing efficiency of *Glyma02g13230, Glyma02g13380, Glyma02g13401, Glyma02g13470, Glyma02g13570*, and *Glyma02g13630* compared to that od control estimated by qRT-PCR. Bars indicating the standard errors, and Significant differences were tested by Student’s t-test, *P < 0.05. **(G–J)** Accumulation of SMV strains SC4 and SC20 on the silenced plants ten days after inoculation detected by qRT-PCR and ELISA. G accumulation of SC4 detected by qRT-PCR, H accumulation of SC4 detected by ELISA, I accumulation of SC20 detected by qRT-PCR, and J accumulation of SC20 detected by ELISA. Different letters indicate the significant differences as tested by ANOVA/LSD (*P* < 0.05).

We used BPMV-based VIGS vectors to validate the functions of six SC4 and SC20 candidate resistant genes (*Glyma02g13230, Glyma02g13380, Glyma02g13401, Glyma02g13470, Glyma02g13570*, and *Glyma02g13630*) in Kefeng-1. Silencing vectors were designated as VIGS‐13230, VIGS‐13380, VIGS‐13401, VIGS‐13470, VIGS‐13570, and VIGS‐13630, respectively, and BPMV empty vector (VIGS‐empty) was used as control. These VIGS vectors were propagated on susceptible cultivar NN1138‐2 plants. Unifoliate leaves of Kefeng-1 plants were inoculated with BPMV recombinant viral vectors. After one week, the trifoliate leaves of these VIGS vector-inoculated plants were re-inoculated with the SMV strain SC4. The silencing efficacy of all silenced plants was examined by qRT-PCR and was found to be 74%, 73%, 74%, 65%, 61%, and 58%, respectively (**Figure 5**). SMV accumulation was detected by qRT-PCR and a DAS‐ELISA. Plants silenced with *Glyma02g13380* accumulated more SMV than control plants, whereas those silenced with *Glyma02g13230, Glyma02g13401, Glyma02g13470, Glyma02g13570*, and *Glyma02g13630* did not show any deposits of SMV (**Figure 5**). Therefore, we can conclude that all six candidate genes were effectively silenced, and *Glyma02g13380* could be the most likely gene mediating resistance against SMV strains SC4 and SC20 in Kefeng-1.

### 3.6 *Glyma02g13380* in Kefeng-1 was distinct from the genes in Dabaima and Qihuang-1

**Figure 6:**
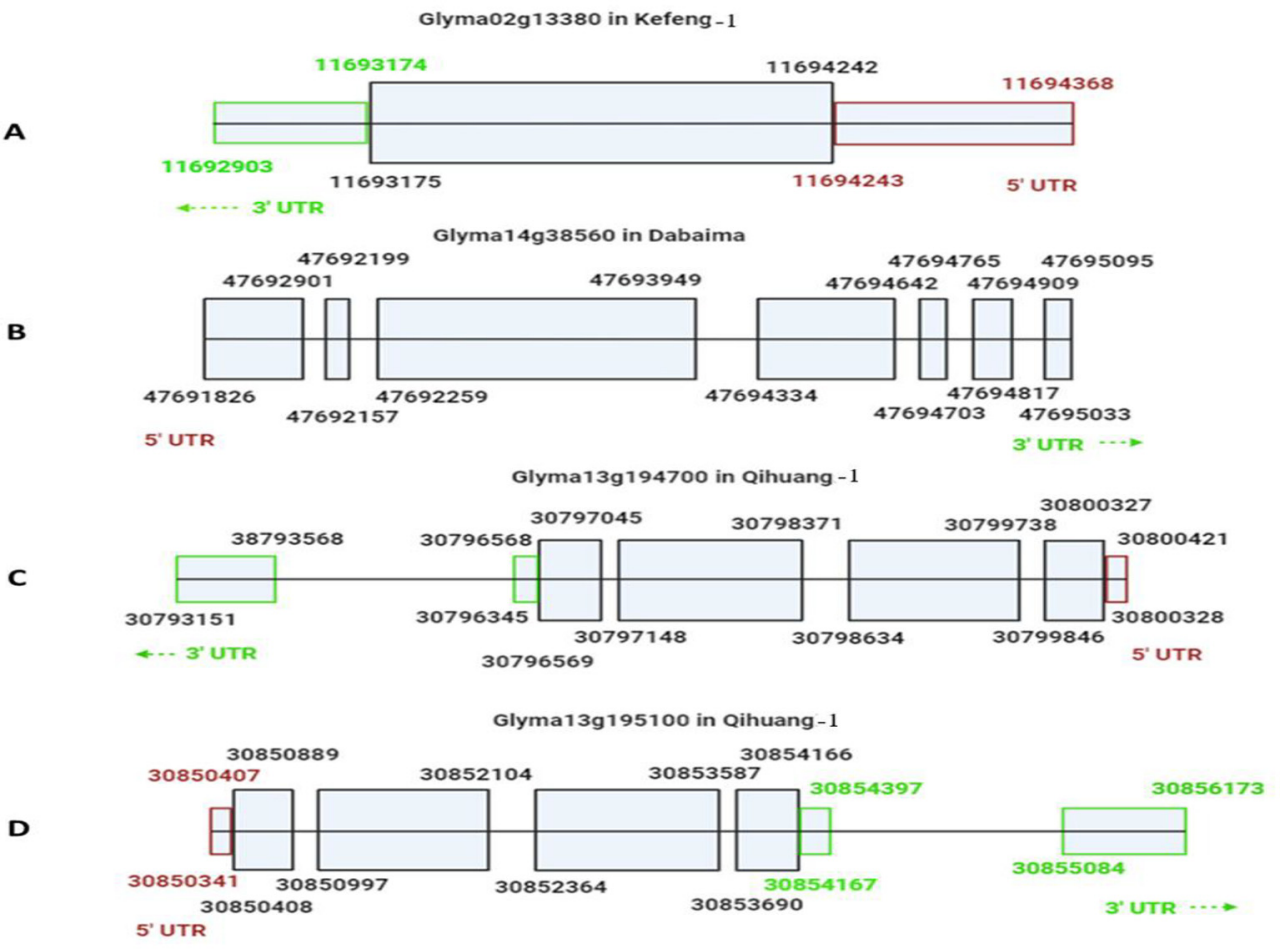
Drafted models of candidate resistant genes for SC4 and SC20 in different Chinese soybean cultivars. **(A)** *Glyma02g13380* responsible for SC4 resistance in Kefeng-1 showed one exon from 11693175 bp to 11694242 bp, 3’UTR from 11692903 bp to 11693174 bp (green color), and 5’UTR 11694243 bp to 11694368 bp (purple color). **(B)** *Glyma14g38560* was responsible for SC4 resistance in Dabaima, as determined by Wang et al. (2011) with a long coding sequence 47691826 bp-47695095 bp divided into seven exons of different sizes. **(C)** *Glyma13g194700* and **(D)** *Glyma13g195100* for SC20 in Qihuang-1 determined by Karthikeyan et al. (2018) with coding sequences of 30796569 bp–30800327 bp and 30850408 bp–30854166 bp, respectively, each divided into four exons.

Our candidate genes for SC4 and SC20 resistance in Kefeng-1 appeared to be dissimilar to the previously identified candidate resistance genes in Dabaima and Qihuang-1. However, to confirm this, we used the cloned coding sequences of SC4 and SC20 resistance candidates described by Wang et al. (2011) and Karthikeyan et al. (2018) and compared them with our candidate genes (**Supplementary Figure 1**). We also drafted models of the candidate genes according to the SoyKB (http://soykb.org). *Glyma02g13380* had a single exon of 1068 bp whereas the coding sequences of the other candidates were divided into several exons of different lengths (**Figure 6A**). The 2538 bp coding sequence of *Glyma14G38560* had seven exons (**Figure 6B**). *Glyma13g194700* and *Glyma13g195100* both exhibited a 3288-bp sequence and were divided into four exons (**Figures 6C, D**).

From the sequence and gene model comparisons, it is clear that *Glyma02g13380*, which is resistant to SC4 and SC20 in Kefeng-1, identified in the current study, was a completely different gene from the already identified candidate genes for these strains in Dabaima and Qihuang-1, and no evidence of translocation or mutation was found. As a result, it can be concluded that *Glyma02g13380*, a joint candidate gene for SC4 and SC20 resistance in Kefeng-1, is not a result of translocation or mutation of an already identified candidate but can be characterized as a distinct gene. Therefore, the two Mendelian genes on Chr.2 in Kefeng-1 for SC4 and SC20 resistance appear to represent a unique common gene, different from *R_SC4_* in Dabaima and *R_SC20_* in Qihuang-1. Furthermore, two more genes might be involved in a single strain resistance.

## 4 Discussion

### 4.1 Genetic mechanism of two separate genes conferring the resistance to the same strain

In Mendelian inheritance, a type of interaction is known between two genes, designated as duplicate genes. Hence, a trait conferred by the two genes interacted with their phenotypic segregation ratio in F_2_ as 15 dominants: 1 recessive. This type of inheritance was explained by the two genes being duplicated, with their phenotypic expression being dominant for either one or two dominant alleles in their genetic construction. In the present study, we identified two genes, one on Chr.14 in Dabaima and one on Chr.2 in Kefeng-1, conferring the same resistance to SC4; however, the sequences of the two genes were different. Similar results were obtained for SC20. In both cases, the two independent genes were not identical. We are unsure whether this was an inheritance of duplicate genes. To explain this, we need to determine whether any particular sequence with both genes on Chr.14 and Chr.2 is responsible for SC4 resistance to conduct an inheritance study. The same is true for SC20. Alternatively, we need to find a reasonable hypothesis for duplicate genes, whether they may be different but exhibit the same function, or whether they should be strictly duplicated. Therefore, the present results do not describe a pair of duplicate genes. However, as mentioned earlier, this requires further study.

### 4.2 An efficient procedure in fine-mapping SMV resistance gene through SNPLDB genome-markers integrated with WGRS gene-markers

In fine mapping, a gene, usually derived from a subpopulation such as the residual heterozygote-derived population, was used. The preparation of the derived subpopulation is time-consuming. Taking advantage of genome-wide sequencing, linkage maps have been constructed using binary markers that cover the whole genome to locate genomic regions associated with specific SMV strain resistance. A genetic linkage map with high-resolution visibility is a powerful tool for identifying genomic regions with specific traits (Karthikeyan et al., 2017). In this study, two linkage maps were constructed using SNPLDB genome markers integrated with WGRS markers for each resistance gene. First, 3683 SNPLDB markers were used to build a high-density resolution map covering all 20 chromosomes of *Glycine max* to preliminarily map the SC4 and SC20 resistance genes. Second, the WGRS gene marker data were used to further locate SNP(s) in the coding region of the resistance gene in both strains. These are efficient techniques for fine-mapping candidate gene(s) against specific SMV strains. The linkage map of 3683 SNPLDB markers built on 427 RILs revealed that the genomic region of 253 kb size affiliated with SC4 resistance was located between SNPLDB320-SNPLDB321 and the 375 kb genomic region associated with SC20 on Chr.2 (LGD1b), whereas the WGRS gene-marker map located the SC4 and SC20 loci between SNPs 11692903 and 14449821 with a shorter distance to 11692903.

The candidate genomic regions for SC4 and SC20 resistance that we uncovered on Chr.2 in the current study resembled many other reported intervals on Chr.2 and Chr.13 for SMV resistance. Zhao et al. (2016) discovered a 30.8-kb candidate resistance region on Chr.2 for *R_SC8_*, and Li et al. (2015) discovered an 80-kb candidate resistance region on Chr.2 for *R_SC18_* in the cultivar Kefeng-1. Karthikeyan et al. (2017) found a 500-kb region for *R_SC5_* on Chr.2 in the cultivar Kefeng-1. Similarly, on Chr.13, possible resistance regions for specific SMV strains have been identified in many soybean cultivars, such as Qihuang-1, Suweon97, and Pl96983 (Karthikeyan et al., 2018; Ma et al., 2016; Yang et al., 2013). These experiments and results can serve as supportive indices for the relevance of WGRS of the SNPLDB and gene marker mapping techniques for the determination of candidate resistant genes using a population of 427 recombinant inbred lines.

### 4.3 Functional validation of disease-resistant candidate genes via BPMV VIGS Vectors

Vectors based on bean pod mottle virus (BPMV) have been used extensively for functional studies of soybean genes (Zhang et al., 2010). After identifying and characterizing genes for specific traits, it is necessary to validate their functions. VIGS is a fast, efficient, and low-cost post-transcriptional gene silencing (PTGS) technique for investigating the functions of target genes (Senthil and Mysore, 2011). In the past, most VIGS vectors used for gene silencing were RNA-based viruses that have been used to silence many types of host crops, including (hot pepper, sweet pepper, Arabidopsis, Nicotiana, cotton, rice, barley, and corn) to remove intrinsic transcripts (Kant and Dasgupta, 2017). Traditional VIGS systems have been effectively employed to explore how plants respond to biotic and abiotic stressors (Shi et al., 2021). Boevink et al. (2016) employed VIGS to determine the mode of action of a novel fungal effector chemical in *Phytophthora infestans* (the pathogen responsible for late blight disease in the Solanaceae family) (Boevink et al., 2016). Keeping in mind the aforementioned successful functional validation of disease resistance genes, we also validated our candidate gene resistant to SMV in the soybean cultivar Kefeng-1 using the BPMV vector via virus-induced gene silencing and confirmed that *Glyma02g13380* is the most likely gene conferring resistance against SC4 and SC20 in Kefeng-1.

### 4.4 Non-nucleotide binding site-leucine-rich repeats (NBS-LLR) also confer resistance against SMV

Resistance to SMV in soybean is very complex, varies from cultivar to cultivar and strain to strain (Hajimorad et al., 2018), and is controlled by different genes belonging to different families. Mostly, genes belonging to the NBS-LRR gene family confer resistance against SMV and other diseases (Cao et al., 2020; Ji et al., 2020; Song et al., 2019; Wei et al., 2023). However, non-NBS-LRRs and unknown genes from different families have also been found to confer resistance to SMV (Khatabi et al., 2012; Wang et al., 2011). For example, one of the SC4 candidate resistance genes, *Glyma14g38580* in Dabaima (Wang et al., 2011) belongs to the P450 gene family, which is known to play multiple roles, such as regulation of growth and development processes, biosynthesis of secondary metabolites, and early defense against diseases in plants (Vasav and Barvkar, 2019). Similarly, two SMV isolates of strain G2 were highly similar in their coding sequences but differed in their virulence against SC4 resistance. This difference in virulence can be attributed to a single amino acid change in the P3 protein of the non-LLR gene (Khatabi et al., 2012). Almost all candidate genes conferring resistance to *Rsv4* on Chr.2 in soybeans are non-NBS-LRRs (Ishibashi et al., 2019). *Glyma02g13380* candidate resistance gene against SMV strains SC4 and SC20 in the current study is also uncharacterized and considered a non-NBS-LRR gene in soybean (https://www.soybase.org). Moreover, the homolog of this gene (AT1G69160) in *Arabidopsis thaliana* is involved in auxin transport (GO:0060918) and positive regulation of the auxin-mediated signaling pathway (GO:0010929). Auxins move into, out of, or within a cell, or between cells, with the help of mediators, such as transporters or pores. The auxin-mediated signaling pathway involves a series of molecular signals generated in response to auxin detection. Auxins are chiefly involved in plant growth and development, but their involvement in defense responses has also been suggested. The function of auxin incentive genes under biotic stress conditions is regulated by their differential expression in rice (Ghanashyam and Jain, 2009).

## 5 Conclusion

We identified and fine-mapped *Glyma02g13380*, a new single SC4 and SC20 resistance gene, on chromosome 2 in Kefeng-1 using GBS and SNPLDB fine-mapping approaches. The inheritance results confirmed that the resistance to SC4 and SC20 in Kefeng-1 was controlled by a single dominant gene. The function of this gene was validated using qRT-PCR, VIGS, and ELISA. The qRT-PCR and ELISA results confirmed that SMV was deposited only on plants that were silenced with *Glyma02g13380* but not on the control and other plants. Thus, *Glyma02g13380* was identified as a new candidate resistance gene against SC4 and SC20 in Kefeng-1.

## Data Availability

The original contributions presented in the study are included in the article/**Supplementary Material.** Further inquiries can be directed to the corresponding authors.

## Supplementary material

Supplementary Table 1 Responses of 10 differential hosts to Soybean Mosaic Virus strains SC4 and SC20

Supplementary Table 2 qRT-PCR primers list with sequences, size and annealing temperature

Supplementary Table 3 Primer with their sequences for gene fragments confirmation, silencing efficiency and CP contents.

Supplementary Table 4 Functional annotation of seventeen candidate genes in genomic region related to SC4 and SC20 resistance

Supplementary Figure 1 Sequence alignment of resistant candidates for SC4 and SC20. *Glyma02g13380* in Kefeng-1 (this work), *Glyma14g38560* in Dabaima, and *Glyma13g194700* and *Glyma13g195100* in Qihuang-1.
