# Supplementary material for: Identification and functional validation of a new gene conferring resistance to Soybean Mosaic Virus strains SC4 and SC20 in soybean
Source: Front Plant Sci. 2025 Jan 27;15:1518829. doi: 10.3389/fpls.2024.1518829 (PMC11811538; doi:10.3389/fpls.2024.1518829)
Supplement: Supplementary Table 1 — Responses of 10 differential hosts to Soybean Mosaic Virus strains SC4 and SC20 [file Table1.docx]

**Supplementary Table 1** Responses of 10 differential hosts to *Soybean Mosaic Virus* strains SC4 and SC20

| **Strain** | **NN 1138-2** | **Youbian 30** | **8101** | **Tiefeng 25** | **Davis** | **Buffalo** | **Zaoshu 18** | **Kwanggyo** | **Qihuang-1** | **Kefeng-1** |
| --- | --- | --- | --- | --- | --- | --- | --- | --- | --- | --- |
| SC4 | -/M | -/M | -/M | -/M | -/- | -/- | -/- | -/- | -/- | -/- |
| SC20 | -/M | N/N | -/M | -/- | -/N | N/N | N/N | -/- | -/- | -/- |

Note: Reaction of inoculated leave/reaction of upper leave; -/- =symptomless; M=mosaic; N=necrosis

**Supplementary Table 2** qRT-PCR primers list with sequences, size and annealing temperature

| **Primer name** | **Primer Sequence** | **Size(bp)** | **Temp. (℃)** |
| --- | --- | --- | --- |
| Glyma02g13230-F | CACGAAGAAGCTCTCCAACG | 168 | 60 |
| Glyma02g13230-R | AGGATCGGAATCGAGGAACC | 168 | 60 |
| Glyma02g13361-F | GCTGCGAAACAGACTTGCTA | 116 | 60 |
| Glyma02g13361-R | CTCCCATGACAGATGGTGGT | 116 | 60 |
| Glyma02g13371-F | AGAGACAGCCGAGTTTCCAA | 146 | 60 |
| Glyma02g13371-R | AGAGCATGACCAACACTGGA | 146 | 60 |
| Glyma02g13380-F | CAGAAGCTCAAGCACTGCAGAT | 172 | 60 |
| Glyma02g13380-R | GCTGAAAAGGACAGTGCATGCTTG | 172 | 60 |
| Glyma02g13401-F | AACCGTGGCAAGCTGTATGAGTTC | 197 | 60 |
| Glyma02g13401-R | TGCTGTAGTGCCTCAACTCTGGAT | 197 | 60 |
| Glyma02g13470-F | ATGCTGACACCAAGTTCCTGGTAG | 155 | 60 |
| Glyma02g13470-R | AGCTGGGTAGAAGTGAAGGCGAAT | 155 | 60 |
| Glyma02g13570-F | CACATGGAGTCAGCTGATTG | 66 | 56 |
| Glyma02g13570-R | CATCCTCAGGATTCCAAGGT | 66 | 56 |
| Glyma02g13630-F | GGTTTACCGTGTGGTGCTTT | 188 | 56 |
| Glyma02g13630-R | TGTTATCACCGTGTGCCTCT | 188 | 56 |
| Tubulin-F | TTCGTGGAGGTCAGCTTCTT | 129 | 60 |
| Tubulin-R | TCCAGGATGCAAGCTGGTTA | 129 | 60 |

**Supplementary Table 3** Primer with their sequences for gene fragments confirmation, silencing efficiency and CP contents.

| **Sr. No.** | **Primers** | **Sequences** |
| --- | --- | --- |
| 1 | BPMV-R2-C2F | TGACATTCTCCTGGGAATTTCCC |
|  | BPMV-R2-C2R | CACACTTCACACATCATTACGAC |
| 2 | Glyma02g13230-qF | CGGGTTCGGGAATCTAGACG |
|  | Glyma02g13230-qR | AGACGACCATGATGCCACTG |
| 3 | Glyma02g13380-qF | CACCACCACCACCTACACTC |
|  | Glyma02g13380-qR | CAAGCCTTCCACCAGGAGAG |
| 4 | Glyma02g13401-qF | TAGGGGAAAGGTGGAGCTGA |
|  | Glyma02g13401-qR | TCAAGTGTCTTGGCCATGCT |
| 5 | Glyma02g13470-qF | TGCGGGTCCAACGTATCAAA |
|  | Glyma02g13470-qR | ACTTGGTGTCAGCATCGGAG |
| 6 | Glyma02g13570-qF | AACTGGCACTGTTCGTCACA |
|  | Glyma02g13570-qR | TATCCTTCCACCACCGTTGC |
| 7 | Glyma02g13630-qF | ATTGCTGGATCCCAGTCTGC |
|  | Glyma02g13630-qR | ACCTTCCCAAACTCCAACCC |
| 8 | Tubulin-F | GGAGTTCACAGAGGCAGAG |
|  | Tubulin-R | CACTTACGCATCACATAGCA |
| 9 | SMV-CP-qF | TTCTGAAAGTCCGTATATGCCTAG |
|  | SMV-CP-qR | GCCTTTCAGTATTTTCGGAGTT |

**Supplementary Table 4** Functional annotation of seventeen candidate genes in genomic region related to SC4 and SC20 resistance

| **Sr. No** | **Gene name** | **Homologs in Arabidopsis** | **Gene annotation** |
| --- | --- | --- | --- |
| 1 | *Glyma02g13230* | AT1G75310 | auxin-like 1 protein |
| 2 | *Glyma02g13240* | AT1G69210 | Uncharacterized protein family UPF0090 |
| 3 | *Glyma02g13250* | AT1G13700 | 6-phosphogluconolactonase 1 |
| 4 | *Glyma02g13260* | AT1G69200 | fructokinase-like 2 |
| 5 | *Glyma02g13270* | AT4G30000 | Dihydropterin pyrophosphokinase / Dihydropteroate |
| 6 | *Glyma02g13280* | AT1G13690 | ATPase E1 |
| 7 | *Glyma02g13290* | AT1G31690 | Copper amine oxidase family protein |
| 8 | *Glyma02g13310* | AT5G38970 | brassinosteroid-6-oxidase 1 |
| 9 | *Glyma02g13320* | AT3G24240 | Leucine-rich repeat receptor-like protein kinase family protein |
| 10 | *Glyma02g13330* | AT3G08900 | Reversibly glycosylated polypeptide 3 |
| 11 | *Glyma02g13340* | AT4G36470 | S-adenosyl-L-methionine-dependent methyltransferases supper family protein |
| 12 | *Glyma02g13350* | AT1G69180 | Plant-specific transcription factor YABBY family protein |
| 13 | *Glyma02g13361* | AT4G38440 | Uncharacterized protein |
| 14 | *Glyma02g13371* | AT1G69170 | Squamosa promoter-binding protein-like (SBP domain) transcription factor family protein |
| 15 | *Glyma02g13380* | AT1G69160 | Uncharacterized protein DDB_G0271670-like |
| 16 | *Glyma02g13401* | AT5G15800 | MADS-box transcription factor |
| 17 | *Glyma02g13420* | AT1G69120 | MADS box protein |
| 18 | *Glyma02g13450* | AT2G03720 | Universal stress protein family |
| 19 | *Glyma02g13460* | AT3G51550 | Protein tyrosine kinase |
| 20 | *Glyma02g13470* | AT3G51550 | Protein kinase domain |
| 21 | *Glyma02g13495* | AT5G50740 | Copper transport protein atox1-related |
| 22 | *Glyma02g13520* | AT1G69060 | DnaJ domain |
| 23 | *Glyma02g13530* | AT1G69050 | Uncharacterized protein LOC102666246 |
| 24 | *Glyma02g13540* | AT1G69040 | Trna-nucleotidyltransferase/polymerase family member |
| 25 | *Glyma02g13555* | AT4G15090 | Mule transposase domain |
| 26 | *Glyma02g13570* | AT2G03810 | Pre-ribosomal assembly protein gar2-related |
| 27 | *Glyma02g13580* | AT2G03830 | Uncharacterized protein LOC100799293 [Glycine max] |
| 28 | *Glyma02g13590* | AT1G26300 | BSD domain |
| 29 | *Glyma02g13603* | AT5G22860 | Protease s28 pro-x carboxypeptidase-related |
| 30 | *Glyma02g13616* | AT5G22860 | Serine carboxypeptidase S28 |
| 31 | *Glyma02g13630* | AT1G69020 | Prolyl oligo peptidase family |

Functional annotation with information of homologous genes of Arabidopsis obtained from SoyBase (<http://www.soybase.org>) using soybean William 82 as reference genome (GlymaWm82. a1. v1).
