# Supplementary figures and images for: Identification and functional validation of a new gene conferring resistance to Soybean Mosaic Virus strains SC4 and SC20 in soybean
Source: Front Plant Sci. 2025 Jan 27;15:1518829. doi: 10.3389/fpls.2024.1518829 (PMC11811538; doi:10.3389/fpls.2024.1518829)

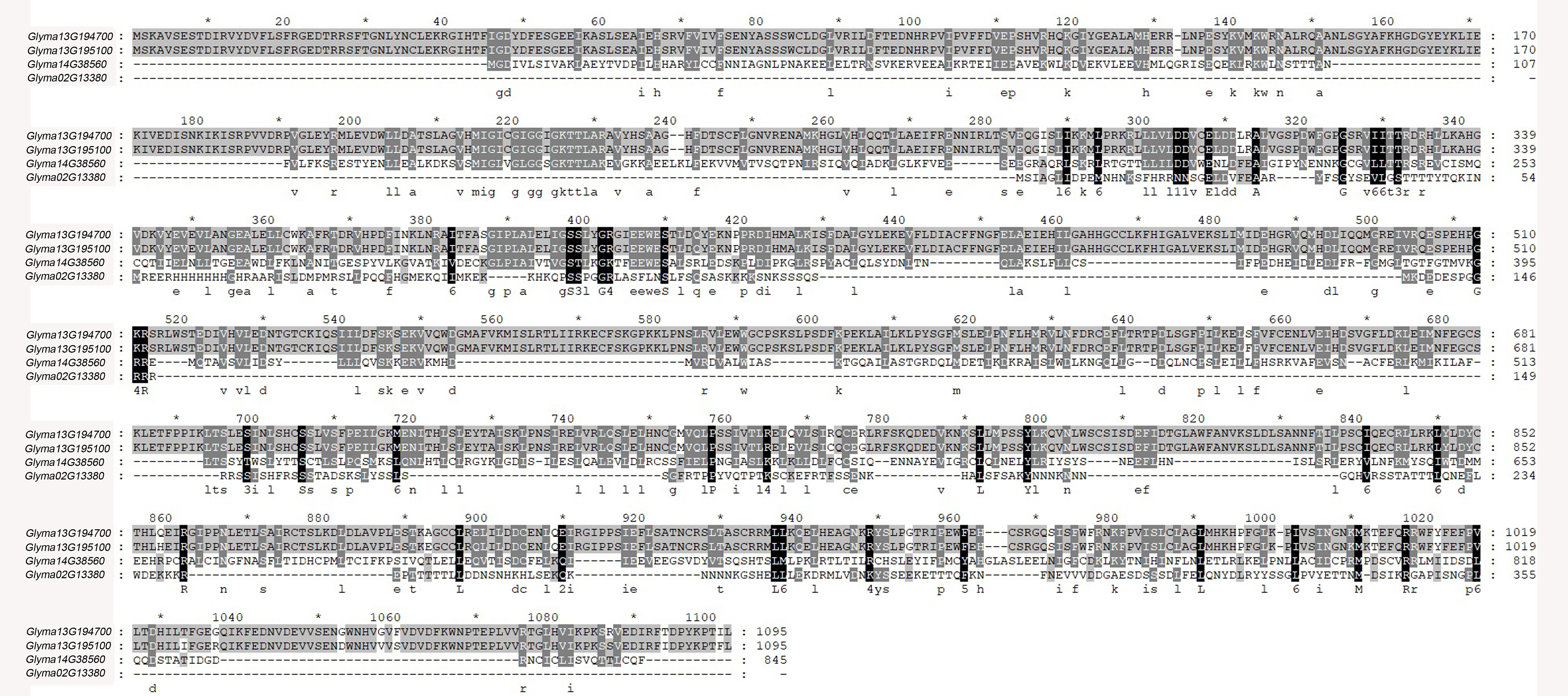

Supplement: Supplementary Figure 1 — Sequence alignment of resistant candidates for SC4 and SC20. Glyma02g13380 in Kefeng-1 (this work), Glyma14g38560 in Dabaima, and Glyma13g194700 and Glyma13g195100 in Qihuang-1. [file Image1.jpeg]
